# Awareness of the use of non-steroidal anti-inflammatory drugs: A cross-sectional study

**DOI:** 10.3892/mi.2025.248

**Published:** 2025-06-16

**Authors:** Lasha Chkhikvadze, Elisabed Chikobava, Nithesh Hariharan, Amritha Omprakash, Syed Mohammed Aamil

**Affiliations:** American MD Program, Tbilisi State Medical University, 0186 Tbilisi, Georgia

**Keywords:** Non-steroidal anti-inflammatory drugs, over-the-counter drugs, electronic survey, knowledge, attitude

## Abstract

Non-steroidal anti-inflammatory drugs (NSAIDs) are widely utilized across the globe and are frequently used without prescription for various therapeutic purposes. Given their potential for severe adverse effects, assessing the awareness of the use of NSAIDs is crucial. The present study evaluated the awareness levels of the use of NSAID among consumers through a descriptive, cross-sectional, non-randomized study. A 33-item online survey was conducted with 385 NSAID consumers aged ≥18 years between August and October, 2023. Awareness levels were determined based on knowledge and attitude scores assigned to correct responses. The results revealed that headaches (62.9%) and menstrual pain (32.4%) were the primary reasons for the use of NSAIDs, with ibuprofen being the preferred generic NSAID. The overall knowledge of NSAIDs was found to be inadequate, particularly concerning associated risks, such as stroke and myocardial infarction, despite a good understanding of the gastrointestinal risks. Female participants, those aged 26-45 years, and individuals with higher education levels demonstrated better knowledge. A concerning tendency toward self-administration and over-the-counter use was observed. These findings highlight the need for targeted educational initiatives and regulatory interventions to improve NSAID awareness and promote responsible medication practices.

## Introduction

Non-steroidal anti-inflammatory drugs (NSAIDs), acknowledged for their pivotal role in modern medicine, are widely used globally. Their effectiveness in providing analgesic, anti-inflammatory and antipyretic benefits is well-established, with ongoing discussions on their potential protective roles against colorectal cancer and cardiovascular conditions (1). Globally, ~30 million individuals use NSAIDs daily, often available over-the-counter (OTC) (2). In Georgia, the NSAID market is extensive, comprising >800 types, with 85% offered OTC, and the remaining 15%, although prescription-only, are readily accessible.

The existence of the ample amount NSAID-containing products and the ready availability of OTC medications have unfortunately contributed to their misuse (3). The World Health Organization highlights a concerning trend of inappropriate prescription and dispensation of medications worldwide, resulting in misuse by almost half of the patients, thereby escalating morbidity and mortality rates (4). Further, pharmacoepidemiologic analyses indicate a growing NSAID usage, potentially contributing to increased toxicity incidents (2). While NSAIDs provide substantial therapeutic advantages, their inappropriate use, such as incorrect dosing, prolonged use, the combined use of multiple NSAIDs, or neglecting proper timing, can lead to various side-effects, ranging from dyspepsia to gastrointestinal ulcerations and bleeding, acute and chronic renal failure, hypertension, myocardial infarction and hemorrhagic stroke (5,6).

The awareness of the use of NSAIDs among consumers has been evaluated throughout the years by several extensive survey studies carried out globally. These studies have consistently demonstrated the widespread usage of NSAIDs, often inappropriately, with consumers largely unaware or unconcerned about potential the NSAID-related toxicities (7-13). The existing evidence suggests a lack of knowledge regarding current patterns of NSAID use and consumer perceptions, markedly affecting medication use behaviors. Despite these global studies, Georgia has not been previously explored in this context. It is crucial to conduct a comprehensive assessment of the public awareness of the use of NSAIDs in Georgia, a developing Eastern European country with an evolving healthcare system facing various challenges.

Through the identification of knowledge and attitude gaps and risk groups for NSAID misuse in Georgia, the present study aimed to provide insight into the healthcare landscape and potential areas for improvement. Additionally, the present study aimed to guide the establishment of impactful public awareness programs that could lessen the hazards related to the use of NSAIDs. This exploration is essential for enhancing healthcare outcomes and preventing complications arising from inadequate awareness.

The present study is novel in its approach, focusing on Georgia, a country with an evolving healthcare system and unique regional challenges, unlike previous studies conducted globally (9-18). The use of large-scale survey data, which were analyzed through robust statistical methods, provides valuable insight into public awareness and attitudes towards the use of NSAIDs, a critical area for improving healthcare interventions. The present study provides new evidence on the knowledge and attitudes towards the use of NSAIDs in Georgia, filling an important gap in the existing literature. The findings, supported by robust data analysis, hold the potential to inform public health policies and interventions, fitting well within the realm of big data applications in healthcare, where large datasets are analyzed to uncover trends, behaviors and actionable insights for improving health outcomes.

## Subjects and methods

### Study subjects and data collection

The Tbilisi State Medical University Biomedical Research Ethics Committee reviewed the protocol prior to data collection and issued ethical authorization in compliance with the principles of the Declaration of Helsinki, on July 21, 2023. The present study adopted a non-randomized, cross-sectional, descriptive and comparative observational approach, with data collected prospectively using a structured questionnaire. The present study focused on Georgian citizens aged ≥18 years, who had used at least one NSAID for any reason during the previous year, examining their awareness and self-reported usage patterns without directly evaluating adherence to standard clinical practice.

The sample size of 385 subjects was calculated using Raosoft Sample Size Calculator (Raosoft, Inc.), an online tool, based on an error margin of 5%, a power of 50%, a confidence interval of 95%, and a Georgian population size of 3,736,400 subjects. Confidence intervals were not included in the tables. Inclusion criteria required participants to be Georgian citizens aged ≥18 years and to have used NSAIDs during the past year, while exclusion criteria included incomplete responses.

A 33-item questionnaire was developed to assess NSAID awareness. It was divided into sections covering sociodemographic details, NSAID use, sources of information, knowledge, attitudes and satisfaction with current NSAID knowledge. The questionnaire items were initially developed in English based on similar studies and then translated into Georgian. A bilingual expert reviewed both versions to ensure linguistic and conceptual accuracy. As the study was conducted in Georgia and targeted a Georgian-speaking population, only the Georgian version was used during data collection. The questionnaire was validated through a pilot study involving 10 medical and 10 non-medical participants, and feedback from this process was used to refine the final version.

The finalized questionnaire was distributed digitally via Google Forms, (Google LLC), an online survey tool, on various social media platforms. Participant recruitment occurred from August 1, 2023, to October 30, 2023. Data were collected in Google Sheets, (Google LLC), an online spreadsheet tool. where responses were anonymized, and access was restricted to the research team to ensure data confidentiality.

The questionnaire included instructions detailing the aims of the study and clarified that it focused exclusively on NSAIDs, explicitly excluding aspirin, paracetamol and other analgesics. The introductory section provided informed consent, explaining the aim, features, future use of the data, confidentiality, potential risks and benefits, and included the contact information of the principal investigator. It also included a checkbox, and by agreeing to the statement ‘I agree to the terms and conditions’, participants provided their consent to participate in the study.

The knowledge section included 14 multiple-choice questions, each with one correct answer contributing one point to the overall knowledge score (range, 0-14). No partial credit was given, and incorrect answers received zero points. The attitude section comprised nine statements assessed using a 5-point Likert scale (always, often, sometimes, rarely and never), with scores ranging from 0 to 4 per item. For positively framed statements (e.g., consulting a doctor prior to NSAID use), higher frequency responses received higher scores. For negatively framed statements (e.g., taking NSAIDs without reading instructions), responses were reverse-scored so that a higher frequency of undesirable behaviors resulted in lower scores. The total attitude score ranged from 0 to 36. A threshold of 60% of the maximum score (i.e., ≥9 for knowledge and ≥22 for attitude) was used to define satisfactory scores.

### Statistical analysis

Statistical analysis was performed using IBM SPSS Statistics 23.0 (IBM Corp.), a statistical software package. Skewness and kurtosis were calculated to assess the normality of the data, and the Shapiro-Wilk test was employed to further evaluate distribution fit. Categorical variables were expressed as frequencies and percentages, while continuous variables were summarized as the mean, median and standard deviations. The unpaired Student's t-test was applied to compare knowledge and attitude scores (presented as the mean ± standard deviation) between males and females, participants with and without chronic disease, and participants with and without medical education, with all comparisons derived from continuous variables. Pearson's correlation analysis was used to assess the correlations between continuous variables, such as knowledge and attitude scores. One-way ANOVA was used to compare scores across different age groups, education levels, and regional residents, followed by Tukey's test as a post hoc analysis to identify specific group differences. A value of P<0.05 was considered to indicate a statistically significant difference.

## Results

### Sociodemographics

A total of 385 completed questionnaires were collected and analyzed (Table I). The participant cohort had an age range of 18-85 years, with a mean age of 43.26 years and a median age of 43 years. A marked proportion of those surveyed were female, comprising up 66.8% of the study sample. Geographically, the majority resided in the largest cities of Georgia, with 22.3% of the subjects residing in Batumi and 37.3% of the subjects residing in Tbilisi, the capital. As regarding the level of education, a notable portion of the subjects, accounting for 36.6%, held a bachelor's degree. The survey also revealed that 25.2% of the participants had a medical background, including MDs, medical students, nurses, dentists, pharmacists and public health workers. Finally, 111 participants (28.8%) reported having a chronic disease.

### Reasons for using NSAIDs

**Figure 1:**
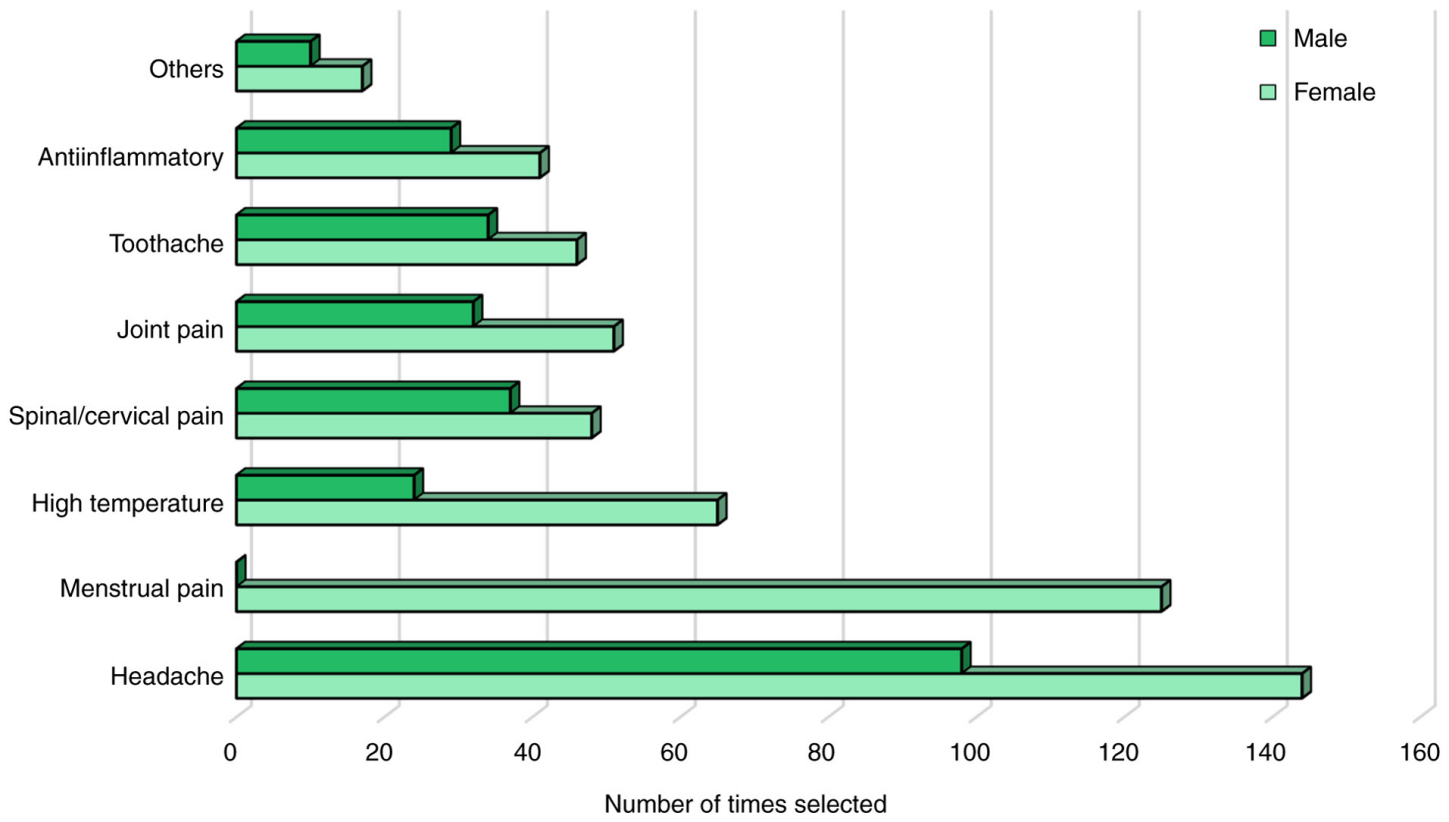
Common reasons for the use of non-steroidal anti-inflammatory drugs among males and females.

The reasons for the use of NSAIDs among the Georgian population were diverse (Fig. 1). The most commonly reported reason was headache, selected by 242 participants (62.9%), including 144 females (59.5% of those who reported headache) and 98 males (40.5%). Menstrual pain was the second most common reason overall, reported by 125 females (48.6% of all females). Notably, among the female participants, headache was more frequently cited than menstrual pain (63.0 vs. 48.6%). Other frequent indications included a high body temperature (n=89; 65 females, 24 males), cervical/spinal pain (n=85; 48 females, 37 males), joint pain (n=83; 51 females, 32 males), toothache (n=80; 46 females, 34 males) and anti-inflammatory purposes (n=70; 41 females, 29 males) (Fig. 1). A sex-based comparison of the shared reasons for the use of NSAIDs (i.e., all indications apart from menstrual pain) did not reveal any statistically significant differences (data not shown). Of all the participants, 173 (44.9%) subjects stated they used NSAIDs for a single reason, 170 (44.2%) for two to three reasons and 42 (10.9%) for more than three reasons.

### Commonly used NSAIDs

**Figure 2:**
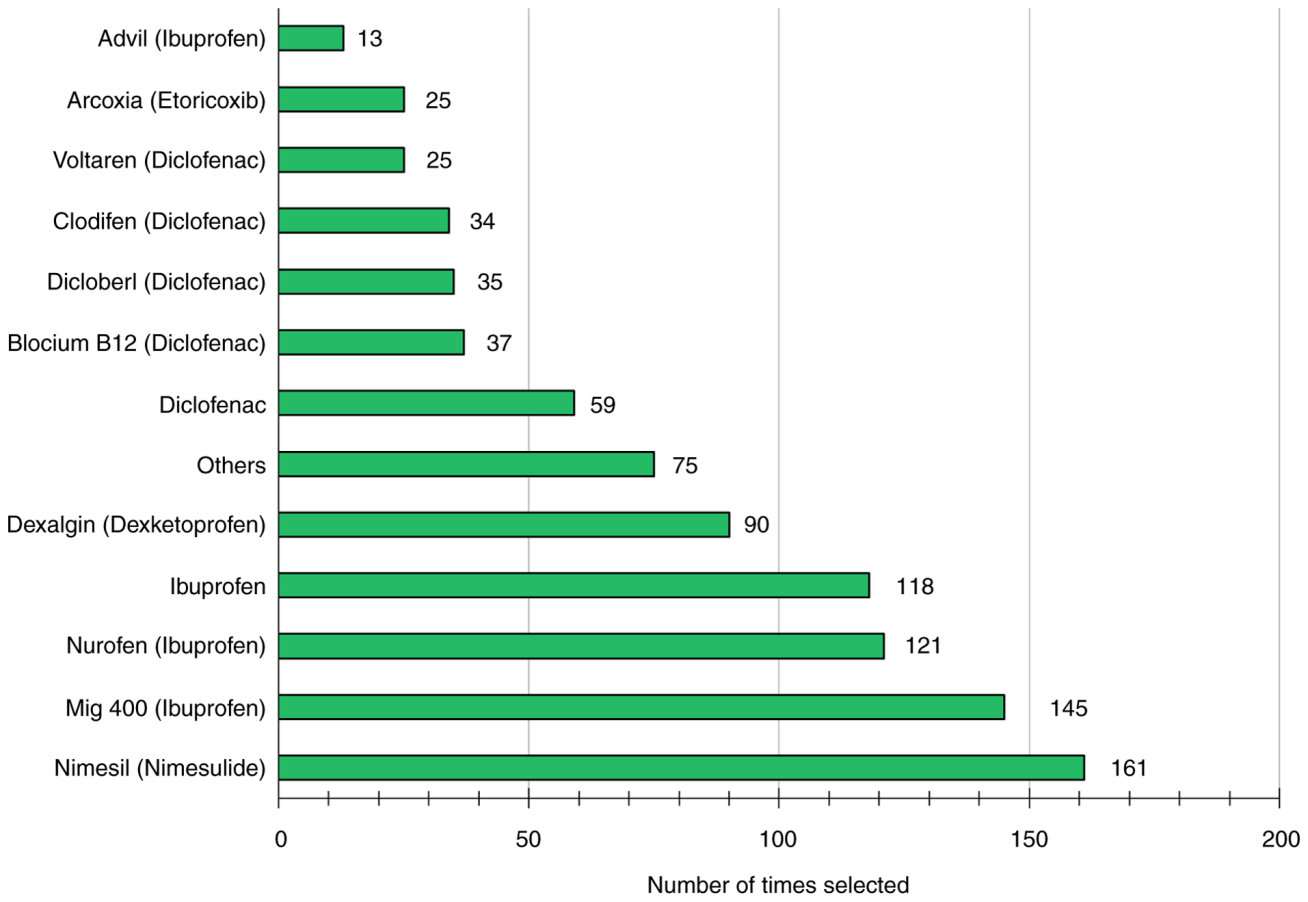
Commonly used non-steroidal anti-inflammatory drugs.

The patterns of NSAID usage in Georgia revealed distinct preferences for specific brands and generics (Fig. 2). The most widely used brand-name NSAID was Nimesil (nimesulide), closely followed by Nurofen (ibuprofen) and Mig 400 (ibuprofen). Dexalgin (dexketoprofen) was also very popular on the list.

In terms of generic NSAIDs, the data revealed that ibuprofen was selected 397 times (42.3% of all selections), diclofenac 190 times (20.2%), nimesulide 161 times (17.1%) and dexketoprofen 90 times (9.5%) (Fig. 2). These numbers indicate the frequency of the selection of each generic NSAID, not distinct individual choices. It is crucial to note that these percentages were calculated based on the total instances of selections, which amounted to 938. Participants had the option to select multiple choices, including various brands of ibuprofen. This element is essential for understanding the study's trends in drug selection.

A total of 145 participants (37.7%) reported having used a single type of NSAID, 163 participants (42.3%) reported using two to three types, and 77 participants (20%) reported using more than three types of NSAIDs throughout their lives.

### Sources of information on NSAIDs

**Figure 3:**
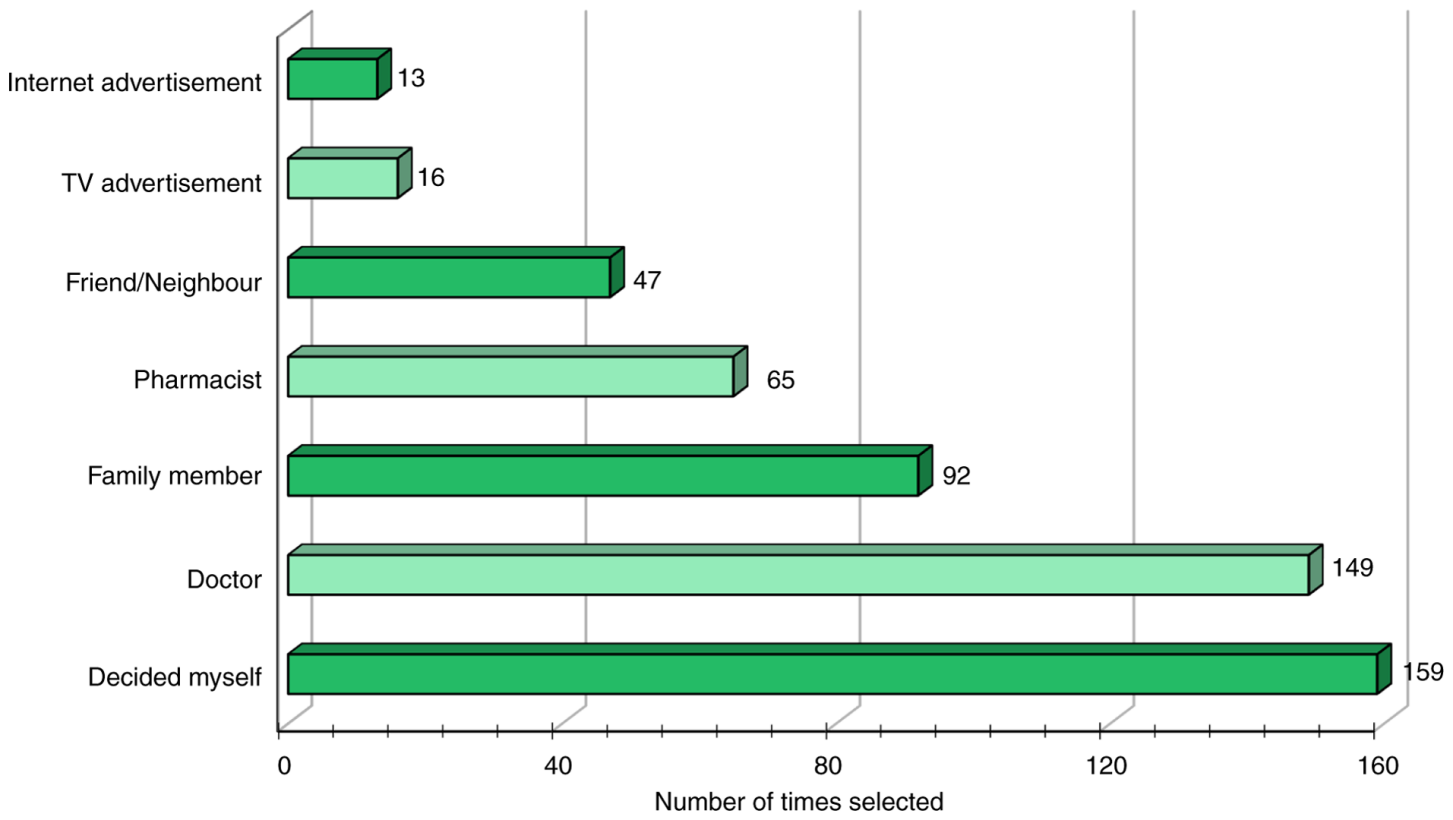
Common sources of advice/information on non-steroidal anti-inflammatory drugs.

Primary sources of information and advice on NSAIDs exhibited variability among the study subjects (Fig. 3). A substantial number of the participants, amounting to 159 (41.2%), engaged in self-medication practices by selecting NSAIDs based on their discretion. It should be noted that only 54 (34%) of the participants had a medical background. On the other hand, 149 (38.7%) of the participants complied with the advice provided by physicians. The selection of NSAIDs was also impacted by family members' advice for 92 participants (23.8%). Furthermore, less common sources of influence included pharmacists, media outlets, and suggestions from friends and neighbors.

### Knowledge regarding the use of NSAIDs

The knowledge level regarding NSAIDs varied among Georgian NSAID users (Table II). Notably, ~70.6% of the study subjects accurately recognized key information, such as taking NSAIDs after a meal and the potential for oral NSAIDs to cause peptic ulcers and gastric irritation. Additionally, a majority displayed an understanding of appropriate timing and storage conditions, while over half of the participants were knowledgeable about the cautionary use of NSAIDs in specific patient categories (pregnant, lactating, asthmatic and hypertensive).

On the other hand, it was noted that the participants knew less about the potential adverse effects of NSAIDs. Only 29.6 and 30.6% recognized the elevated risk of stroke and myocardial infarction, respectively. Furthermore, 39.2% were misinformed about injectable NSAIDs being safer for the stomach, and 47.2% believed NSAIDs could be taken safely alongside other medications.

**Figure 4:**
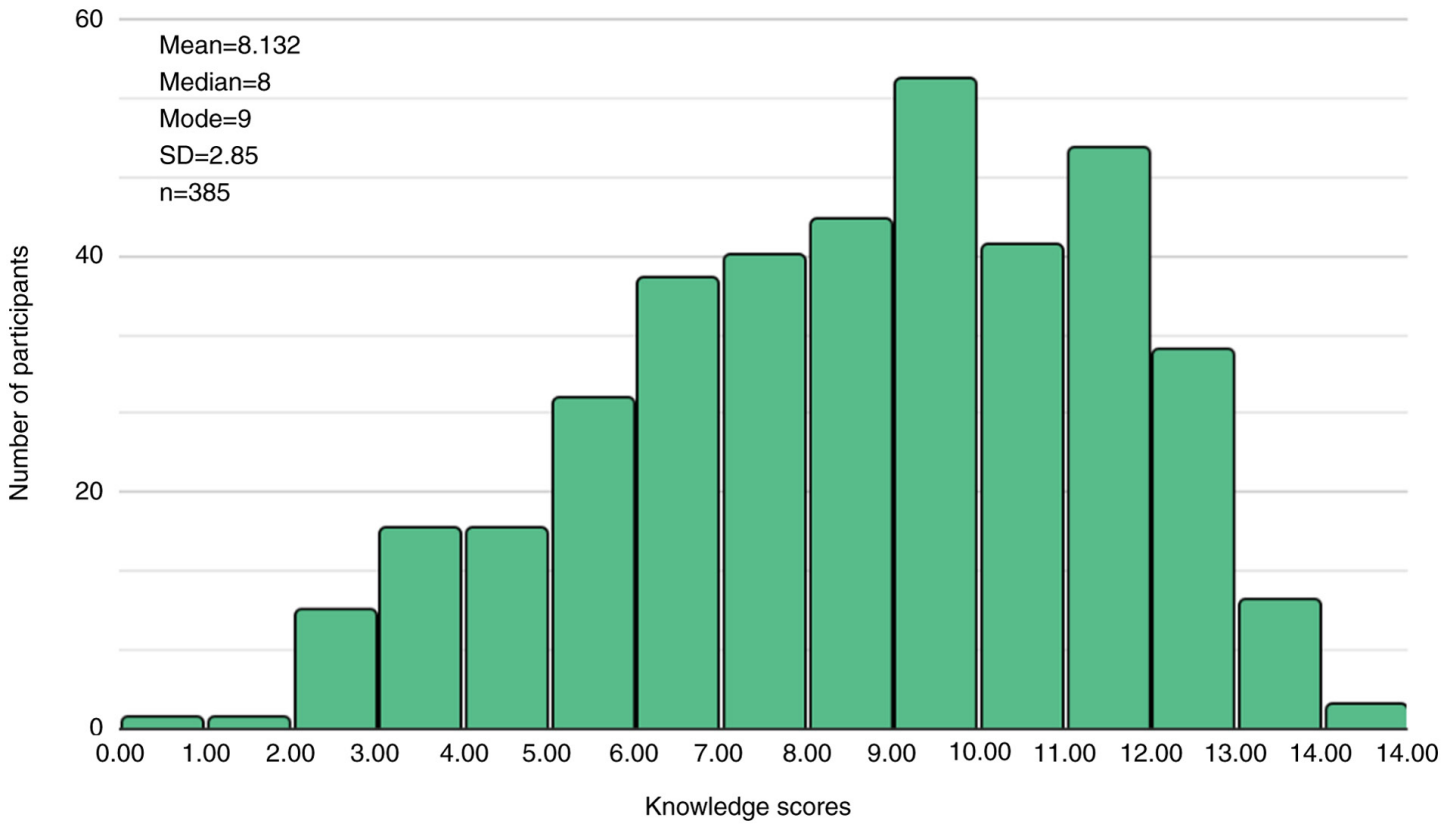
Distribution of knowledge scores.

The mean knowledge score was calculated as 8.132±2.87 points, falling below the satisfactory threshold, with a distribution exhibiting normality (Fig. 4). The statistical analysis yielded significant outcomes. The mean total knowledge score of the female participants was significantly higher (8.87±2.34) than that of the male participants (6.47±3.18; P<0.001) (Table III). Additionally, participants in the age groups of 26-35 and 36-45 years demonstrated mean knowledge scores of 8.81±2.81 and 8.48±2.89, respectively, which were significantly higher than those in the 65+ age group (6.59±2.04) (P<0.05,) (Tables IV and V). The mean knowledge score for participants with chronic diseases (8.75±2.94), compared to those without chronic diseases (7.88±2.78), was statistically insignificant (P=0.224) (Table III).

Educational qualifications played a role, with participants holding a Bachelor's degree (8.13±2.88) and PhD (9.36±1.74) exhibiting significantly greater knowledge scores than those with basic or secondary education (6.92±2.71) (P<0.05). Additionally, participants with a Master's degree (8.99±2.44) also demonstrated significantly higher knowledge scores compared to those with basic or secondary education (6.92±2.71) (P<0.001) (Tables VI and VII). Furthermore, those with a medical background scored considerably higher on the knowledge assessment (9.29±2.45) than non-medical participants (7.74±2.88) (P<0.001) (Table III).

### Attitudes and behaviors regarding the use of NSAIDs

The study subjects displayed diverse attitudes and behavioral patterns concerning the use of NSAIDs (Table VIII). A substantial number of participants stated always (49.9%) or often (22.1%) consuming NSAIDs for new types of pain without seeking a doctor's consultation. In addition, 77.9% of the participants purchased NSAIDs from pharmacies over the counter. Although 169 (43.9%) of those surveyed stated they always took NSAIDs after meals, very few of them regularly also took gastroprotectors. Encouragingly, the majority of the participants stated that they always or often checked the expiration date and read the instructions provided for NSAIDs prior to use, respectively. Furthermore, almost half of the interviewees said they always or often carried NSAIDs with them.

**Figure 5:**
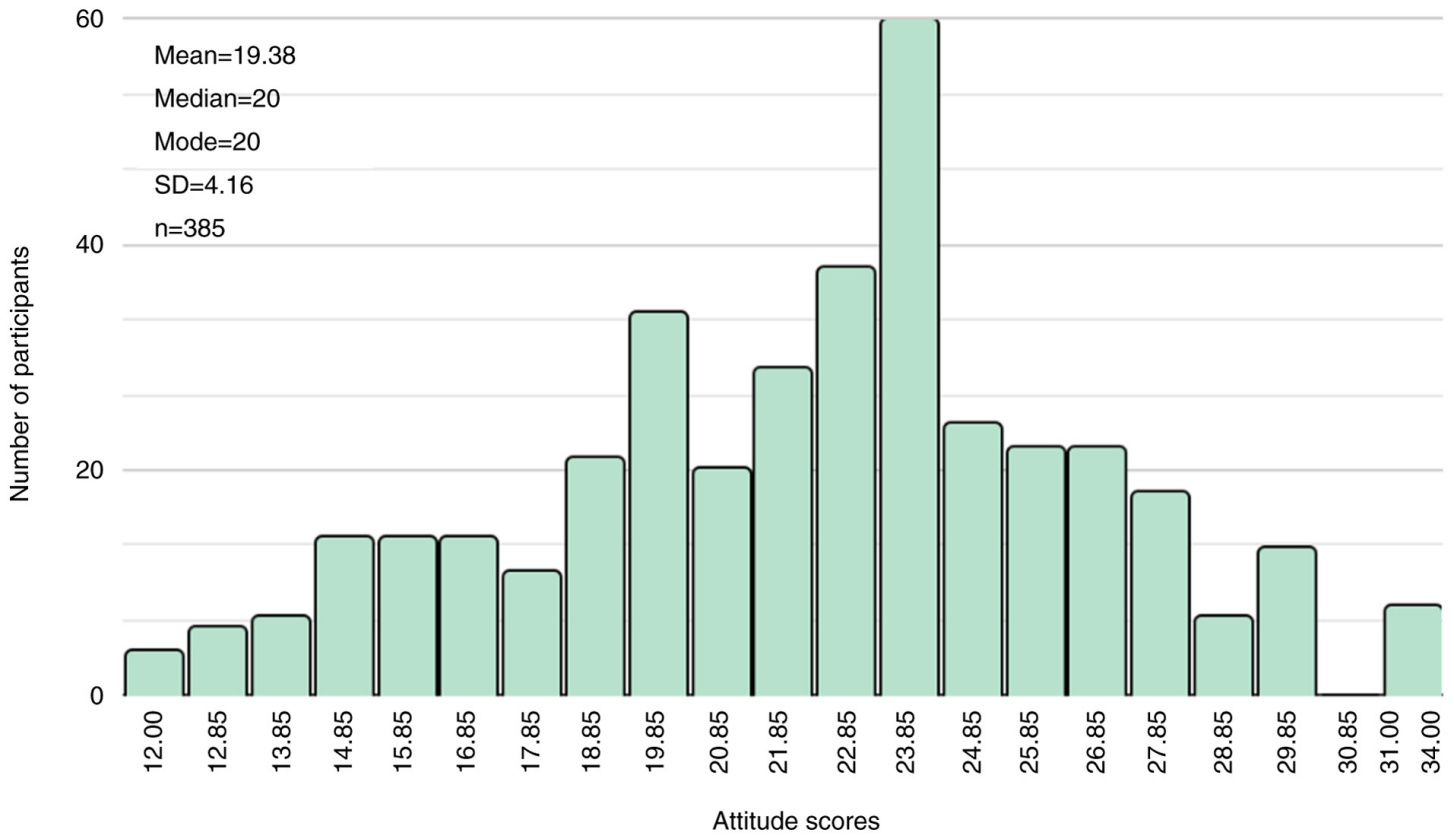
Distribution of attitude scores.

The mean attitude score of the participants was calculated as 19.38±4.16 points, with a range from 0 to 36, which also falls below the satisfactory threshold. The distribution of attitude scores among the study subjects exhibited a normal distribution (Fig. 5).

**Figure 6:**
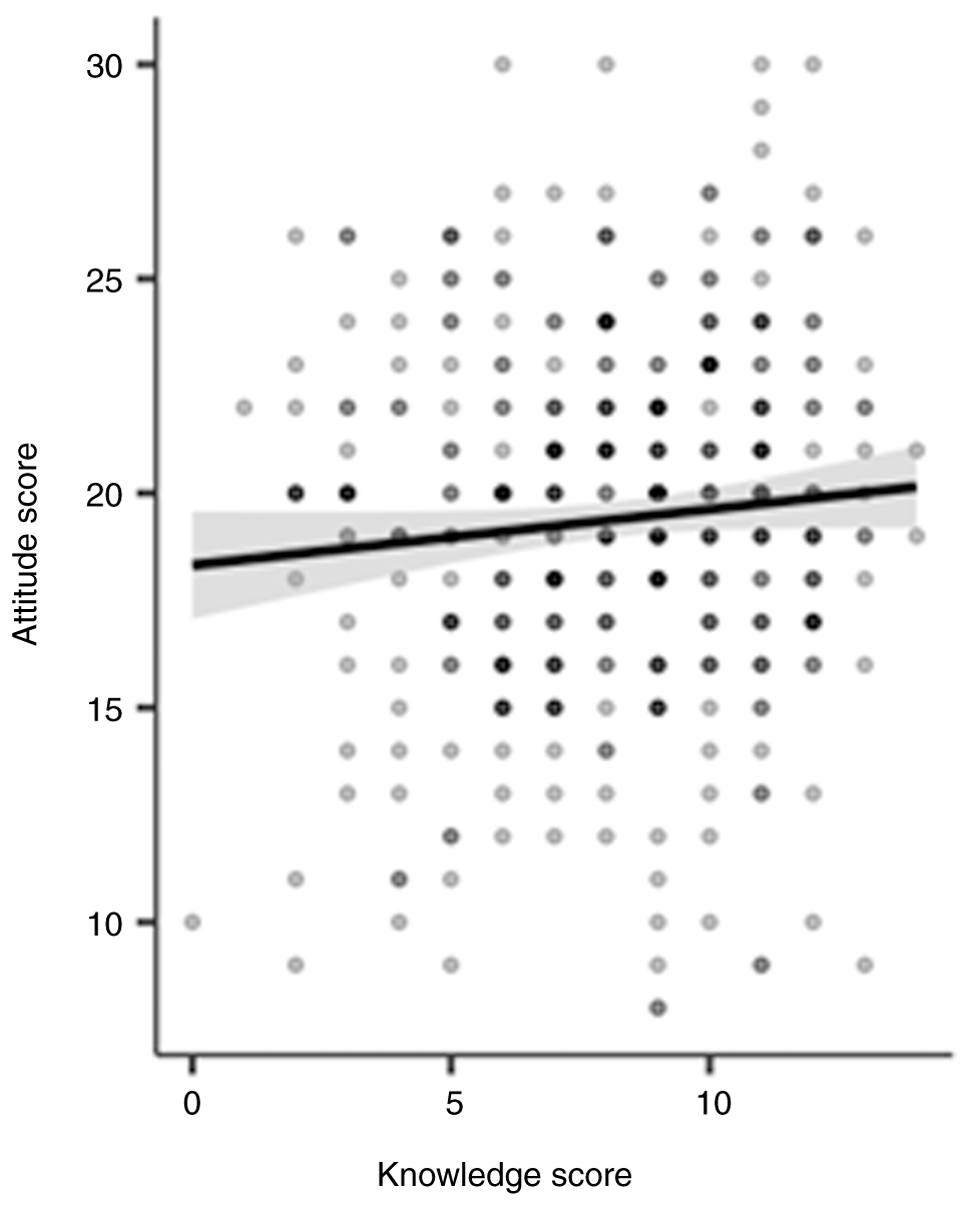
Correlation plot between knowledge and attitude scores.

While statistically significant associations were observed for knowledge scores, no such associations were found between attitude scores and other factors. The mean attitude score for participants with chronic diseases (22.6±4.55) was similar to that of participants without chronic diseases (22.3±4.29) (P=0.708,) (Table III). Participants with a medical education had a mean attitude score of (22.7±4.38), while those without a medical education scored (22.34±4.35) (P=0.690,) (Table III). For sex comparisons, the mean attitude score for females was (22.6±4.37), compared to (22.1±4.34) for males (P=0.717, (Table III). These differences were not statistically significant. Additionally, Pearson's correlation analysis revealed a weak positive correlation between knowledge and attitude scores (P<0.05,), suggesting that as participants' knowledge of the use of NSAIDs, their attitudes toward their use tends to improve slightly (Table IX and Fig. 6).

Lastly, a substantial proportion of the study participants, 296 (76.9%), stated dissatisfaction with their current NSAID knowledge and exhibited a willingness to learn more.

## Discussion

The present study provided valuable insight into the awareness of NSAIDs among Georgians in 2023, marking the first study of its kind to assess knowledge, attitude, and behavioral specificities towards the use of NSAID in this developing Eastern European country. Online surveys were employed to ensure that the study sample was not limited to any particular subgroup by allowing a wide geographic reach throughout Georgia. Notably, only a few international studies have utilized similar online methods so far (7-14). The present study, as with numerous other similar studies conducted globally, predominantly involved female participants (8-10,14-17). In order to overcome the limitations observed in previous studies, characterized by the inadequate representation of diverse age groups and educational levels, the present study strategically shared the survey across various social media groups to enhance visibility and involvement across all demographics. Consequently, these aspects contributed to a higher degree of generalizability to our study's findings.

The findings of the present study indicated ibuprofen as the most frequently selected generic medication, representing 42.3% of all instances of selection. This observation aligns with patterns identified in comparable international studies (7,8,10,14,16,18,19). Diclofenac and dexketoprofen closely followed, which also exhibit popularity in studies conducted in various countries (10,11,16,18). However, ketoprofen was less popular in Georgia, while it is popular in Albania and Italy (9,16).

While ibuprofen stood out as the dominant generic NSAID, Nimesil (nimesulide) emerged as the dominant brand of NSAIDs in Georgia, a unique observation not found in similar international studies. Nimesulide is distinct due to its basic sulfonanilide structure, providing prompt analgesic, antipyretic and anti-inflammatory effects by selective COX-2 inhibition (20). However, it is crucial to recognize that the global usage of nimesulide is limited due to concerns about hepatotoxicity compared to other NSAIDs (21,22). Notably, it has never undergone evaluation by the FDA and is unavailable in the USA, UK, Canada, Australia, New Zealand, Japan, Finland, Spain, Ireland and Singapore (23,24). The European Medicines Agency acknowledged the potential risks, assessed its benefit-risk balance, and permitted its restricted use, mainly for acute pain and primary dysmenorrhea, excluding osteoarthritis treatment (25). Local regulations in Georgia require a doctor's prescription for purchasing Nimesil. Nevertheless, the results of the present study demonstrate extensive over-the-counter availability, which may be attributed to the commercial interests of pharmacies and inadequate governmental oversight. Notably, Nimesil in Georgia is available in 100 mg/2 g granule packets, frequently sold individually, usually without accompanying written instructions, leaving consumers without adequate guidance and potentially at risk of misuse. Leaving the issue of the over-the-counter availability if nimesulide and the misuse unaddressed in Georgia could lead to severe public health consequences, necessitating urgent and decisive action.

While the present study did not assess the factors influencing NSAID brand preference, it is likely that multiple variables contribute to the preferences of patients. These may include drug pricing, availability across pharmacies, promotional discounts, advertising, route of administration, perceived efficacy and onset of action. For example, the popularity of Nimesil may be partially attributed to its oral granule formulation, rapid symptom relief and widespread availability in single-dose sachets. Overall, NSAID selection in Georgia appears to be multifactorial, and understanding the weight of each factor warrants further investigation.

In the present study, the most common reason for the use of NSAIDs was headache (62.9%). By contrast, studies conducted in Turkey found that while the prevalence of headache was similarly high, musculoskeletal complaints were more common (11,26). In another study in Bahrain, almost 90% of the participants utilized NSAIDs for headaches, and a notable 64% for flu, a pattern not observed in the present study (10). By contrast, in Albanian statistics, flu was the most common cause (21.6%), with headaches being less common (18.1%) (9). The majority of international studies, predominantly involving reproductive-age females, seldom report menstrual pain as a prevalent reason for the use of NSAIDs. In the present study, the online survey revealed a 32.4% use of NSAIDs for menstrual cramps, similar to another study in Saudi Arabia (32%) (14). By contrast, Jordan (23%) (15) and Bahrain (24.7%) (10) reported lower rates through self-administered surveys. Other face-to-face or paper-based interviews often omitted menstrual pain, even though NSAIDs are objectively popular and effective for dysmenorrhea (27,28). This may imply a hesitance to discuss menstrual pain in direct interviews, emphasizing the suitability of online or self-administered surveys for sensitive topics. These results reveal key regional differences in the use of NSAIDs, underlining the need to tailor patient education and healthcare practices accordingly.

The present study in Georgia revealed that 41.2% of the participants self-medicated, similar to Italian (44.7%) Albanian (56.3%), Saudi Arabian (71%), and numerous other international studies (9,14,16,29-31). Doctors as a source of advice/information on NSAID were almost identical in Georgia (38.7%) and Bahrain (38%), in contrast to lower rates in Saudi Arabia (21.2%) and Albania (14%) (9,10,12). Family members were mentioned as a source of information by 23.8% of the Georgian participants, which was greater than Albania (10%), but lower than Australia (32.2%) (9,17). Compared to Georgia (16.8%), Bahrain, Australia, Italy and Saudi Arabia have higher levels of reliance on pharmacists' guidance (10,12,16,17). Unlike other countries, internet advertisements were less popular in Georgia, which was surprising, as there is an increased tendency to use the internet as a source of medical information observed worldwide (12,17,32,33). It may reflect mistrust in online sources or a lack of internet proficiency among the Georgian population, warranting further investigation. This information underscores the importance of considering regional variations in healthcare behaviors and the potential influence of cultural factors.

The present study demonstrated that Georgians have a solid understanding of proper use and storage, with 70.6% correctly identifying the necessity of post-meal NSAID consumption, as in the case of Saudi Arabia (14). By contrast, awareness of this fact was noted in only 31.5% of the participants in studies conducted in Amman, Jordan and 42.4% in Urbana, IL, USA (8,12). Additionally, Georgians displayed better awareness of the potential asthma exacerbations and skin allergies due to NSAIDs, compared to Jordan, Saudi Arabia and Turkey (11,14,15). In addition, the present study revealed a marked awareness of oral NSAID-related gastrointestinal side-effects among Georgian participants, mirroring findings from a number of international studies (7,8,10,11,13,15). This increased awareness of gastrointestinal side effects may be due to individual encounters with symptoms, such as heartburn and epigastric discomfort, as noted in Turkish studies, as well as the high prevalence of these side-effects overall (6,11). While the present study did not delve into personal NSAID side-effect experiences, future research on this prevalence in Georgia could illuminate the reasons behind such informed awareness. Of note, a notable misconception among 39.2% of the participants, believing injectable NSAIDs to be safer for the stomach, was identified. This disparity highlights a specific area for improvement in public understanding.

Awareness of NSAID-induced nephrotoxicity in the present study was relatively low at 48.8%, similar to various nations, but less than Thailand (60%) and Urbana, IL, USA (79.9%) (7-13). The survey also identified knowledge gaps about the risks of strokes (29.6%) heart attacks (30.6%) and NSAID drug-to-drug interactions (47.2%), similar to international pattern (11,12,14,15). Conversely, participants from Georgia had a greater awareness of the hazards associated with hypertension (56.6%), as in Urbana, IL, USA (57.9%) and higher than that of Albania (31.7%) and Turkey (7.1%) (8,9,11).

These findings may indicate a narrower understanding of NSAID dangers in the Georgian population, implying that the seriousness of NSAIDs may have been underestimated. Similar to findings in previous international studies, the present study revealed that the overall knowledge about NSAIDs among Georgians is inadequate, particularly about side-effects (7,8,12-14,30). Females exhibited a better understanding of NSAIDs compared to males, coordinating with findings from various study findings from Norway, the USA, Italy, the UK, Saudi Arabia, and Thailand (13,14,34-37). Sex differences in pain perception may account for this disparity in NSAID knowledge, with women maybe needing more analgesics (38,39). Generally, males are less likely to ask doctors or pharmacists questions, take painkillers, or inquire about drugs (8,16,40,41). On the other hand, women become more accustomed to NSAIDs when they take them for menstrual cramps monthly. Exploring the impact of women's caregiving roles on their NSAID familiarity could be a compelling avenue for future research.

According to the present study, the age group of 26-45 years had the greatest knowledge about NSAIDs, significantly surpassing the 65+ age group, which had the lowest knowledge. Few studies included a sufficient amount of 55+ age groups to make comparisons. Elderly individuals who were relatively younger were shown to be more knowledgeable in a Czech study that focused on members of retirement communities (19). By contrast, a Saudi Arabian study found that individuals <35 years of had less knowledge (14). It is crucial to examinet the reasons behind this knowledge disparity between nations. It could be influenced by differences in healthcare system structure and accessibility, varying emphases on health education, or reliance on diverse information sources such as the internet or healthcare professionals. Given the 96% prevalence of NSAID use in geriatric patients in general practice, the low knowledge of NSAIDs in Georgia is alarming (42). This age group is more susceptible to NSAID-related side-effects due to altered pharmacokinetics, polymorbidity and polypharmacy, increasing the risk of adverse effects and drug interactions compared to younger adults. To address this, focused educational programs and clearer NSAID labeling and regulations may aid in increasing awareness and reducing risks for the elderly (43).

A significant correlation between NSAID knowledge and level of education was found in the present study; those with Bachelor's, Master's, or PhD degrees scored higher than those with only primary or secondary education. This pattern is consistent with research from the Czech Republic, Thailand and Turkey (11,13,19). The gap in NSAID knowledge is particularly concerning as 51% of Georgians only have an elementary or intermediate education, meaning they may have a higher chance of NSAID misuse (44).

In the present study, the overall attitude score concerning NSAID use was below the satisfactory threshold. This aligns with the observed high rate of over-the-counter NSAID consumption and limited consultation with doctors. Notably, the majority of participants reported always or often reading drug instructions and expiration dates, albeit at a lower rate than in Australia, Italy, Saudi Arabia, and Urbana, Illinois, USA (8,14,16,17). The discrepancy between a high frequency of reading instructions and a low level of knowledge regarding NSAIDs may indicate misunderstandings about their adverse effects due to inadequate conversations with healthcare professionals and insufficient attention to label information. Although a majority of participants took NSAIDs after meals and acknowledged the potential for serious gastrointestinal side effects, the notable observation of low usage of gastroprotective agents aligns with findings in the USA, where a significant portion of participants do not take preventive measures to avoid NSAID side-effects (7). A weak yet statistically significant positive correlation was found between knowledge and attitude scores, suggesting that a greater knowledge was associated with a slightly more favorable attitudes. This in contrast to with findings from Turkey, where a stronger alignment between knowledge and attitude was reported (11).

The relatively weak correlation observed in the present study may be attributed to several factors. The knowledge items assessed objective, fact-based understanding, such as timing, side-effects and interactions, whereas attitude items reflected self-reported behavior and beliefs, including consulting doctors or reading instructions. The latter were rated using a Likert scale and may be influenced by social desirability or recall bias, reducing their objectivity. Participants may overreport positive health behaviors or struggle to accurately recall NSAID-related habits. Additionally, this result may reflect a broader behavioral trend in which knowledge does not automatically translate into improved attitudes or responsible use, particularly in habitual or culturally normalized practices such as self-medication with NSAIDs. These findings emphasize the importance of targeted educational interventions that go beyond information delivery and address behavioral motivation and risk perception.

To enhance NSAID awareness in Georgia, focused interventions are necessary. These include comprehensive educational campaigns focusing on proper use, potential adverse reactions, and the necessity of consulting medical professionals, targeting all demographics, with special attention to males, geriatric patients, and individuals with lower education levels. Doctors and pharmacists in particular should take a proactive role in educating patients about the use of NSAIDs and associated risks. With the prevalence of over-the-counter consumption and reliance on product instructions, it is imperative to emphasize digital and alternative information sources. Additionally, regulatory measures for stricter NSAID sales control and clearer labeling, particularly for products with higher misuse risks, are vital. Moreover, the majority of research participants indicated a desire to learn more about NSAIDs, which underscores the necessity of the previously described awareness-raising activities.

The present study has inherent limitations associated with its online survey approach. First, the requirement for internet access and basic digital literacy may have excluded individuals lacking these resources, potentially skewing the sample toward more technologically engaged participants. Additionally, the non-randomized, voluntary nature of the survey introduces the possibility of self-selection bias, as individuals who chose to participate may differ systematically from the broader population. The absence of an interviewer may have led to misinterpretation of certain questions, as participants were unable to seek clarification. Furthermore, the online format carries the risk that participants may have searched for answers while completing the questionnaire, potentially affecting the accuracy and honesty of their responses. Lastly, while the study focused exclusively on Georgian citizens to address a specific gap in local data, this national focus may limit the generalizability of the findings to other populations or settings.

In conclusion, the present study, which represents a noteworthy first in examining knowledge, attitudes, and behavioral patterns toward NSAIDs in Georgia, provides crucial insight into NSAID awareness in this region. The results underscore the necessity of tailored educational approaches and healthcare interventions, particularly given the variations in NSAID awareness among different demographics. On the regulatory level, we recommend stricter enforcement of existing prescription-only rules for NSAIDs, mandatory inclusion of printed instructions even for single-dose sales, and standardized risk warnings on packaging. Further oversight of pharmacy-level dispensing practices is also needed to limit unsupervised access to high-risk NSAIDs. To ensure safer NSAID use in Georgia, our research emphasizes the significance of raising public health awareness and implementing regulatory measures.

## Figures and Tables

**Table I:** Sociodemographic data of the study participants (n=385).

Characteristics	Frequency (%)
Age (years)	
18-25	69 (17.9)
26-35	83 (21.6)
36-45	88 (22.9)
46-55	75 (19.5)
56-65	41 (10.6)
>65	29 (7.5)
Sex	
Male	128 (33.2)
Female	257 (66.8)
Educational status	
Primary-secondary	77(20)
Bachelor	141 (36.6)
Master	94 (24.4)
PhD	14 (3.6)
Professional education	59 (15.3)
Medical background	97 (25.2)
Without a medical background	288 (74.8)
Area of residence	
Tbilisi	145 (37.3)
Batumi	86 (22.3)
Other regions	154(40)

**Table II:** Distribution of the responses of participants to overall knowledge score parameters.

Parameters that determine the overall knowledge score	Frequency (%) of correct answers given
Timing	
Can NSAIDs be taken every 2-3 h when experiencing pain?	267 (69.3)
Can NSAIDs be taken before, during, or after meals?	272 (70.6)
Storage	
Where do NSAIDs need to be stored?	260 (67.5)
Adverse effects	
Do NSAIDs have the potential to cause kidney failure?	188 (48.8)
Do oral NSAIDs cause gastric irritation or peptic ulcer disease?	272 (70.6)
Are NSAIDs administered by injection safe for the stomach?	151 (39.2)
Do NSAIDs have the potential to cause liver failure?	149 (38.7)
Do NSAIDs increase the risk of heart attack?	118 (30.6)
Do NSAIDs increase the risk of brain stroke?	114 (29.6)
Can NSAIDs cause skin rash, itching, swelling, or redness?	224 (58.1)
Cautions	
Is it safe for patients with hypertension to take NSAIDs?	218 (56.6)
Are NSAIDs safe during pregnancy and breastfeeding?	236 (61.3)
Is it safe for asthma patients to take NSAIDs?	195 (50.6)
Drug interactions	
Do NSAIDs interact with certain other drugs when taken together?	182 (47.2)
NSAIDs, non-steroidal anti-inflammatory drugs.	

**Table III:** Comparison of knowledge and attitude scores across participant groups.

Participant group	Knowledge score (mean ± SD)	P-value	Attitude score (mean ± SD)	P-value
Sex				
Female	8.87±2.34	<0.001	22.6±4.37	0.717
Male	6.47±3.18		22.1±4.34	
Chronic disease				
With chronic disease	8.75±2.94	0.224	22.6±4.55	0.708
Without chronic disease	7.88±2.78		22.3±4.29	
Medical education				
With medical education	9.29±2.45	<0.001	22.7±4.38	0.690
Without medical education	7.74±2.88		22.34±4.35	

**Table IV:** Knowledge score distribution among different age groups.

Age groups, years	N	Mean	SD	SE
56-65	41	7.37	3.03	0.473
46-55	75	8.09	2.75	0.317
36-45	88	8.48	2.89	0.308
65+	29	6.59	2.04	0.380
26-35	83	8.81	2.81	0.308
18-25	69	8.03	2.88	0.347

**Table V:** Tukey's post hoc test analysis for knowledge score distribution among different age groups.

		Age groups, years					
Age groups, years	Difference and P-value	56-65	46-55	36-45	65+	26-35	18-25
56-65	Mean difference	-	-0.727	-1.111	0.780	-1.441	-0.6631
	P-value	-	0.797	0.371	0.792	0.123	0.868
46-55	Mean difference		-	-0.384	1.507^a^	-0.714	0.0643
	P-value		-	0.954	0.037	0.591	0.999
36-45	Mean difference			-	1.891^b^	-0.330	0.4483
	P-value			-	0.003	0.974	0.928
65+	Mean difference				-	-2.221^c^	-1.4428
	P-value				-	<0.001	0.068
26-35	Mean difference					-	0.7782
	P-value					-	0.550
18-25	Mean difference						-
	P-value						-

^a^P<0.05,

^b^P<0.01,

^c^P<0.001.

**Table VI:** Knowledge score distribution among different educational levels.

Education level	N	Mean	SD	SE
Bachelor	141	8.13	2.88	0.243
Secondary education	77	6.92	2.71	0.309
Professional education	59	8.07	3.24	0.421
PhD	14	9.36	1.74	0.464
Master	94	8.99	2.44	0.252

**Table VII:** Tukey's post hoc test analysis for knowledge score distribution among different educational levels.

		Education level				
Education level	Difference and P-value	Bachelor	Secondary education	Professional education	PhD	Master's
Bachelor	Mean difference	-	1.21^a^	0.0599	-1.23	-0.862
	P-value	-	0.019	0.999	0.510	0.137
Basic and secondary education	Mean difference		-	-1.1457	-2.44^a^	-2.067^b^
	P-value		-	0.121	0.022	<0.001
Professional education	Mean difference			-	-1.29	-0.922
	P-value			-	0.522	0.268
PhD	Mean difference				-	0.368
	P-value				-	0.991
Master's	Mean difference					-
	P-value					-

^a^P<0.05,

^b^P<0.001.

**Table VIII:** Distribution of participants' responses to overall attitude score parameters.

Parameters that determine the overall attitude Score	Always, frequency (%)	Often, frequency (%)	Sometimes, frequency (%)	Rarely, frequency (%)	Never, frequency (%)
I take NSAIDs for new pain or discomfort without consulting a doctor	192 (49.9)	85 (22.1)	45 (11.7)	49 (12.7)	14 (3.6)
I carry NSAIDs with me	116 (30.1)	83 (21.6)	42 (10.9)	55 (14.3)	89 (23.1)
I take NSAIDs after a meal	169 (43.9)	88 (22.9)	86 (22.3)	32 (8.3)	10 (2.6)
Before using NSAIDs, I check their expiration date	231(60)	47 (12.3)	39 (10.1)	44 (11.4)	24 (6.2)
Before using a new NSAID, I read the instructions	159 (41.3)	70 (18.2)	49 (12.7)	57 (14.8)	50(13)
I take NSAIDs with herbal or food supplements	44 (11.4)	94 (24.4)	41 (10.6)	70 (18.2)	136 (35.3)
In case one NSAID is ineffective, I add a second, different NSAID	51 (13.3)	97 (25.2)	61 (15.8)	76 (19.7)	100(26)
I take a gastroprotection when using NSAIDs	89 (23.1)	88 (22.9)	49 (12.7)	64 (16.6)	95 (24.7)
I buy painkillers over the counter at the pharmacy	300 (77.9)	47 (12.2)	14 (3.6)	13 (3.4)	11 (2.9)

NSAIDs, non-steroidal anti-inflammatory drugs.

**Table IX:** Pearson's correlation analysis for knowledge and attitude scores.

	Attitude score	
Knowledge score	Pearson's R	0.089^a^
	P-value	0.040
	N	385

^a^P<0.05.

## Data Availability

The data generated in the present study may be requested from the corresponding author.
